# Unlocking the Potential of Lignocellulosic Biomass: Microwave and Hydrothermal Pretreatment to Improve the Production of High Value-Added Biorefinery Compounds

**DOI:** 10.1021/acs.energyfuels.5c03953

**Published:** 2025-10-10

**Authors:** Amer Rouabhia, Carlos José Álvarez-Gallego, Luis Alberto Fernández-Güelfo, Mariana Valdez Castillo, Antonio Avalos Ramirez

**Affiliations:** † University of Cádiz, Department of Chemical Engineering and Food Technology, Campus Puerto Real, 11510 Puerto Real, Cádiz, Spain; ‡ University of Cádiz, Department of Environmental Technologies, Campus Puerto Real, 11510 Puerto Real, Cádiz, Spain; § Wine and Food Research Institute (IVAGRO), Campus Puerto Real, 11510 Puerto Real, Cádiz, Spain; ∥ National Center in Environmental Technology and Electrochemistry, Shawinigan, Shawinigan, QC G9N 6V8, Canada; ⊥ Université de Sherbrooke, Department Chemical and Biotechnological Engineering, Faculty of Engineering, Sherbrooke, QC J1N 3C6, Canada

## Abstract

This study is focused on the performance of a hydrothermal reactor (HTR) and microwave-assisted (MW) pretreatments of sugar beet pulp (SBP), orange peel (OP), brewer spent grain (BSG), and rice husk (RH) to evaluate the extraction of high-value biorefinery compounds. The influence of temperature, duration of treatment, and energy consumption on hydrolysis efficiency was evaluated by quantifying total reducing sugars (TRS), proteins (PR), polyphenols (TP), and volatile fatty acids (VFA). MW pretreatment at 180 °C for 30 min yielded 18% TRS and 24% PR from OP, respectively. In contrast, HTR at 200 °C, for 60 min, achieved higher yields of 32% TRS and 22% PR for OP. BSG showed higher responsiveness under HTR, reaching 25% TRS and 20% PR at 220 °C after 120 min. The highest VFA production was 16 g H–Ac/L (BSG, HTR) and 3.2 g H–Ac/L (SBP, MW) after 120 and 5 min at 220 °C, respectively. From the point of view of energy consumption, MW pretreatment consumed significantly less energy (40.1 kJ/g) than HTR (70.85 kJ/g) under equivalent conditions (120 min at 220 °C). In addition, the MW pretreatment proved to be more energy-efficient for simpler substrates (SBP, OP), whereas HTR was optimal for complex biomasses (BSG, RH). Therefore, tailored pretreatment strategies based on substrate type are crucial to optimize energy consumption and maximize bioproduct recovery.

## Graphical abstract

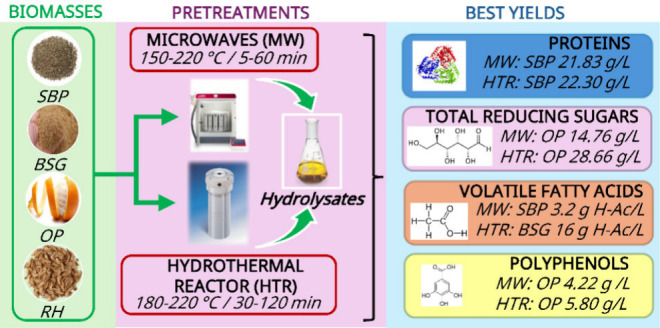

## 1 Introduction

In recent years, biorefinery has attracted significant global interest. Many countries have started to adopt biorefineries as an economic alternative to traditional fossil-based industries for sustainable production and resource management.^1^ The platform concept involves the conversion of biomass into several types of valuable bioproducts^2^ including biofuels, biochemicals, bioplastics, biopharmaceuticals, biocosmetics, bionutrients, biofertilizers, and biomaterials.^3,4^ A pretreatment step is typically integrated into the biorefinery process to enable the efficient fractionation of biomass, which is crucial in the conversion of complex biomasses such as the lignocellulosic materials used in this study. This is often the first stage in a lignocellulosic biorefinery and it is essential for breaking down the complex structure of biomass.^5^ Physicochemical and biological pretreatments have been applied for efficient biomass conversion into valuable bioproducts,^6^ and their selection is based on the specific target products.^7^ Among other pretreatments, hydrothermal pretreatment (HTP) generates great interest because of its simplicity, moderate energy consumption, relatively short processing times and cost effectiveness.^8,9^ In HTP, temperature and pressure play a critical role, typically exceeding 180–200 °C and 15–20 bar. Under these high-temperature and high-pressure conditions, water undergoes increased autoionization, generating hydronium (H_3_O^+^) and hydroxide (OH^–^) ions.^10^ This action mechanism is analogous to the dilute acid pretreatment, enhancing biomass depolymerization and improving the efficiency of downstream processes such as enzymatic hydrolysis and anaerobic digestion.^11^

Under optimal conditions, HTP effectively dissolves hemicellulose and pectin, leading to the releasing valuable byproducts and microbial inhibitors such as xylo-oligosaccharides (XOS), furfural, 5-hydroxymethylfurfural (HMF), acetic acid, levulinic acid, and formic acid.^12^

Indeed, temperatures between 220 and 230 °C are effective for HTP but they pose the risk of degrading released sugars into furfural and HMF, which can be potentially adverse for late biological processes in a biorefinery approach.^13^ In addition, crystalline cellulose undergoes depolymerization at temperatures above 220 °C, while proteins hydrolyze into amino acids between 250 and 400 °C.^14^ These conditions also facilitate lignin depolymerization, producing phenolic compounds such as syringols and catechols.^15^

Parameters such as temperature, pressure, solvent-to-feed ratio, flow rate, solvent type, and operation time play critical roles in determining the efficiency of biomass solubilization and bioproduct recovery.^16^ Furthermore, the choice of heating methods, including electrical heating, microwave radiation, steam injection, or thermal oil systems, can significantly affect the homogeneity and efficacy of hydrothermal pretreatment.

Ruiz et al. (2017) demonstrated the impact of different heating transfer mechanisms (i.e., conduction, convection, and radiation) on process efficiency.^17^ Among those, traditional heating reactor (HTR) and microwave-assisted hydrothermal reactor (MW) systems are the most frequently applied. Microwave-assisted pretreatment offers several advantages including low operational cost, fast processing, and efficient volumetric heating. It requires minimal solvent use and decreases the likelihood of side-reactions.^18,19^ This approach aligns with green chemistry principles, utilizing water as a solvent and biomass as a renewable feedstock, thereby underscoring its potential as a sustainable and effective alternative to traditional heating methods.^20^

The biomass used in this study was chosen based on its global relevance and large availability. Orange peel (OP) is one of the most abundant agro-industrial wastes worldwide, with Spain being the leading citrus producer in Europe generating about 2.65 million tons annually.^21,22^ The orange peel is the waste with the highest volume in the citrus industry. It is estimated that around 20% of the orange is orange peel.^23^ Its strong seasonality can be mitigated by drying and storage. Sugar beet pulp (SBP) is another key byproduct in Spain. Approximately half of the tuber (0.5 kg/kg) is rejected during the industrial process in the form of SBP.^24^ During the 2023/24 agricultural campaign, 3.02 million tons of sugar beets were produced; although seasonal, it is often pelletized for year-round use. Brewer spent grain (BSG), a byproduct of beer production, is continuously generated but undergoes fast microbial degradation because of its high moisture content. Its production was around the 75% of the total byproduct and can be used for biotechnological processes because of its composition.^25^ Finally, rice husk (RH) is produced in more than 150 million tons worldwide (20 kg husk per kg rice). Rice husk contains valuable biomaterials with extensive applications in various fields, and is abundant and relatively easy to store because of its dry and stable nature.^26^

Altogether, these availability patterns and preservation options give potential to the studied biomasses as sustainable feedstocks. This ensures their suitability for industrial-scale biorefinery applications.

From all of the above, this research is focused on a comprehensive comparison between microwave-assisted (MW) and hydrothermal reactor (HTR) pretreatments, aiming to clearly distinguish their effects on product yield, selectivity, and energy consumption. It systematically investigates how different lignocellulosic biomass responds to variations in temperature and operational time, focusing on how these factors influence the distribution and selectivity of valuable bioproducts in the hydrolysate phase. The study also includes a detailed energy consumption analysis, expressed per kilogram of biomass (kJ/g), to evaluate the efficiency and sustainability of each pretreatment method. Furthermore, a statistical analysis was performed to optimize the experimental parameters and validate the significance of the observed trends. To our knowledge, this is the first work to integrate these objectives across multiple biomass types within a single evaluative framework, offering valuable insights into optimizing pretreatment strategies for improved high-value bioproduct production and resource efficiency in biorefinery applications.

## 2 Materials and Methods

### 2.1 Feedstocks and Characterization

In this study, four types of lignocellulosic biomasses were used: orange peel (OP), sugar beet pulp (SBP), brewer’s spent grain (BSG), and rice husk (RH). The OP was sourced from the canteen of the Faculty of Science at the University of Cádiz (Cádiz, Spain). The collected OP was washed several times with distilled water and dried in an oven at 40 °C for 48 h. The SBP was provided by an industrial sugar factory belonging to the AB-Sugar Company located in Jerez de la Frontera (Cádiz, Spain). The RH was obtained from a rice processing plant in Seville (Spain). Both the SBP and RH were obtained as origin-dried material. The BSG was collected from a local craft brewery in Puerto Real (Cádiz, Spain), and the mixture was dried at 60 °C in an oven for 24 h. Following a milling and sieving process, the dried biomass was reduced to a particle size of 1.7 mm. Then, they were stored in a freezer at 4 °C until use. To clarify the role of these preparatory steps, they were performed exclusively to standardize the particle size and moisture content while preserving the chemical structure of biomasses.

### 2.2 Microwave Pretreatment

A Milestone Flexiwave device with a maximum power of 1900 W was used to carry out the microwave hydrothermal pretreatment. The initial dried biomass concentration was 8% (w/v), and the experiments were developed in a 50 mL Teflon vessel with controlled heating. The set temperatures and operation times were 150, 180, 200, and 220 °C for 5, 15, 30, and 60 min. Temperature control was managed by a noncontact infrared sensor, which accurately regulates temperatures up to 300 °C, depending on the vessel type. To ensure consistent microwave energy delivery throughout the process, the reactor system is also equipped with a high-efficiency air-cooled magneton.

### 2.3 Hydrothermal Pretreatment

The hydrothermal pretreatment was carried out using an acid digestion vessel (Parr Instrument, Model 4744, USA) featuring a 45 mL Teflon polytetrafluoroethylene (PTFE) cup housed within a stainless-steel jacket, with a working volume of 30 mL. The system was equipped with a Scientific Fisher Isotemp vacuum oven (Model 282A) 3500 W aperture for temperature control, in which average temperature error was maintained within ± 5 °C.

The substrate was added in a dried biomass wastewater/water ratio of 8% (w/v). The suspension was heated at 150, 200, and 220 °C, with operation times of 30, 60, and 120 min.

### 2.4 Analytical Methods

The analytical methods used were carried out in accordance with previously published work.^21,27^ They were employed to determine total solids (TS), volatile solids (VS), soluble chemical oxygen demand (sCOD), dissolved organic carbon (DOC), volatile fatty acids (VFAs), total reducing sugars (TRS), pH, total polyphenols (TP), and total proteins (PR). For all analyses, samples were centrifuged at 4,000 rpm for 15 min to remove suspended solids, then filtered through 0.45 μm for sCOD and DOC and 0.22 μm for TRS, VFAs, TP, and PR. All measurements were conducted in triplicate.

The solubilization efficiency of the pretreatment process was determined using eq 1, where the OM_f_ and OM_0_ represent the final and initial concentrations of solubilized organic matter in the samples, expressed as COD, DOC, TRS, PR, TVFA, and TP, respectively.

1 $$Y\;\left(\%\right)=100\times \frac{\left({OM}_{f}-{OM}_{0}\right)}{{OM}_{0}}$$

Additionally, the yield on a weight basis (%) was calculated using the following formula:

2 $$Yield\;\left(Wt\%\right)=\left(\frac{Mass\;of\;\mathrm{Pr}oduct}{Mass\;of\;Feedstock}\right)\times 100$$

## 3 Results and Discussion

Table 1 presents the physicochemical characterization of the four lignocellulosic biomasses. All the results have been expressed in % (w/w) on a dry matter basis, except for total solid (TS) and volatile solid (VS), which is reported on a wet basis.

**Table 1:** Composition of Biomasses^a^

Parameter	SBP^b^	BSG^b^	RH^b^	OP^b^
VS (g/kg)	739.03 ± 0.2	261.06 ± 0.2	772.04 ± 0.1	195.06 ± 0.1
TS (g/kg)	833.05 ± 0.5	280.01 ± 0.4	915.00 ± 0.0	206.02 ± 0.8
sCOD (g/kg)	10.60 ± 0.2	21.90 ± 0.7	1.29 ± 0.0	41.10 ± 0.4
DOC (g/kg)	4.09 ± 0.1	7.93 ± 0.1	0.52 ± 0.1	10.10 ± 0.0
TVFA (g H–Ac/kg)	0.87 ± 0.0	1.39 ± 0.0	0.03 ± 0.0	0.43 ± 0.0
Total protein (g/kg)	1.40 ± 0.0	1.30 ± 0.0	0.30 ± 0.0	1.40 ± 0.0
Total polyphenols (g/kg)	0.09 ± 0.0	0.08 ± 0.0	0.05 ± 0.0	1.03 ± 0.0
pH (pH units)	4.37 ± 0.1	5.44 ± 0.2	5.54 ± 0.5	4.17 ± 0.3
DOC/sCOD (%)	38.58 ± 1.2	36.20 ± 1.2	40.31 ± 7.8	24.57 ± 0.2
VS/TS (%)	88.72 ± 0.0	93.23 ± 0.1	84.38 ± 0.0	94.68 ± 0.4
NDF-Soluble fibers^c^ (%)	42.20 ± 1.4	38.00 ± 1.1	16.50 ± 1.2	66.80 ± 1.2
Cellulose (%)	21.10 ± 1.4	16.30 ± 0.4	32.85 ± 0.4	15.70 ± 2.2
Hemicellulose (%)	22.50 ± 0.4	33.70 ± 0.5	22.20 ± 0.6	9.11 ± 0.8
Lignin (%)	3.50 ± 0.0	7.01 ± 0.9	14.00 ± 1.0	1.26 ± 0.1
Rest (%)	10.70 ± 1.4	4.99 ± 1.1	14.5 ± 0.3	7.13 ± 0.3

a VS: volatile solids; TS: total solids; sCOD: soluble chemical oxygen demand; DOC: dissolved organic carbon; TRS: total reducing sugars; TP: total polyphenols; PR: total proteins.

b OP: orange peel; SBP: sugar beet pulp; BSG: brewer spent grain; RH: rice husk.

c NDF Soluble fibers: primarily composed of proteins, pectin, starch, and mucilages.

The total polyphenols (TP) and total proteins (PR) were quantified in the aqueous phase to calculate their concentrations after the pretreatments.

The selection of these four biomasses OP, BSG, SBP and RH was driven by their fiber compositions, moisture content, and potential for solubilization. Primary focus was to represent a range of substrates with varying complexities in fiber structure to understand the efficiency of different pretreatment methods. Fiber content analysis, which results are expressed as a percentage of the biomass in dry weight, was performed according to the Van Soest method.^28^

Initially, it was noticed that OP and the BSG have high moisture content (previously to the lab drying procedure), close to 80% and 30% respectively. On the contrary, SBP and RH present low moisture content since they have been previously dried in the industrial plants where they were generated. Regarding their structural composition, RH was characterized by high concentration of lignin and cellulose, 14.0% and 32.8% respectively. Meanwhile, SBP has a low lignin content of 3.50%, but it is rich in (NDF) soluble fiber of 42.2%. BSG displayed a complex structure with 33.7% hemicellulose, 7.0% lignin, 16.3% cellulose, and 38.0% NDF soluble fiber, which includes starch and pectin. However, OP biomass, as a fresh substrate with no industrial processing, retains its natural composition with a remarkably high pectin content of 66.8% and a minimal lignin content of 1.3%. Polyphenols were present at concentrations across all biomasses, except OP, which contained 1.0 g/kg. The total protein content was relatively high in OP and SBP, 1.4 g/kg and 1.3 g/kg, respectively.^27^ Furthermore, the VS/TS ratio, representing the proportion of organic (volatile) solids relative to total solids, provides insight into the biomass potential for bioconversion. Among the four biomasses, OP shows the highest organic content at approximately 94.65%, followed closely by BSG at 93.23%. SBP and RH have slightly lower organic fractions, 88.69% and 84.34% respectively. This indicates that OP and BSG possess a higher amount of biodegradable organic matter, making them more amenable to pretreatment and subsequent bioprocessing. On the other hand, the DOC/sCOD ratio reflects the quality and biodegradability of the soluble fraction after pretreatment, as (DOC) corresponds to bioavailable carbon, while (sCOD) includes all soluble oxidizable substances. RH presents the highest DOC/sCOD ratio (40.31%), indicating that a relatively larger portion of soluble carbon is bioavailable despite its lower total solubilization. SBP and BSG show similar ratios (38.58% and 36.20%), suggesting a moderate bioavailability of the soluble fraction. In contrast, OP, despite having the highest sCOD value, has the lowest DOC/sCOD ratio (24.57%), implying that a significant portion of its soluble compounds may be less readily biodegradable or more refractory. These diverse fiber and biochemical profiles highlight the significance of selecting a pretreatment method, thereby broadening the applicability of study findings for biorefinery applications.

### 3.1 Statistical Analysis

The statistical analysis method as well as a thorough description of the corresponding methodology can be found in the Supporting Information. In Table S1 are summarized the statistical results, which showed that time and temperature had statistically significant effects on all solubilization parameters, including sCOD, DOC, TRS, VFAs, TP, and PR.

### 3.2 Organic Matter Solubilization

#### 3.2.1 Microwave Assisted Solubilization

The solubilization yield for the biomasses during microwave-assisted (MW) pretreatment was analyzed through sCOD and DOC measurements (Figure 1).

**Figure 1:**
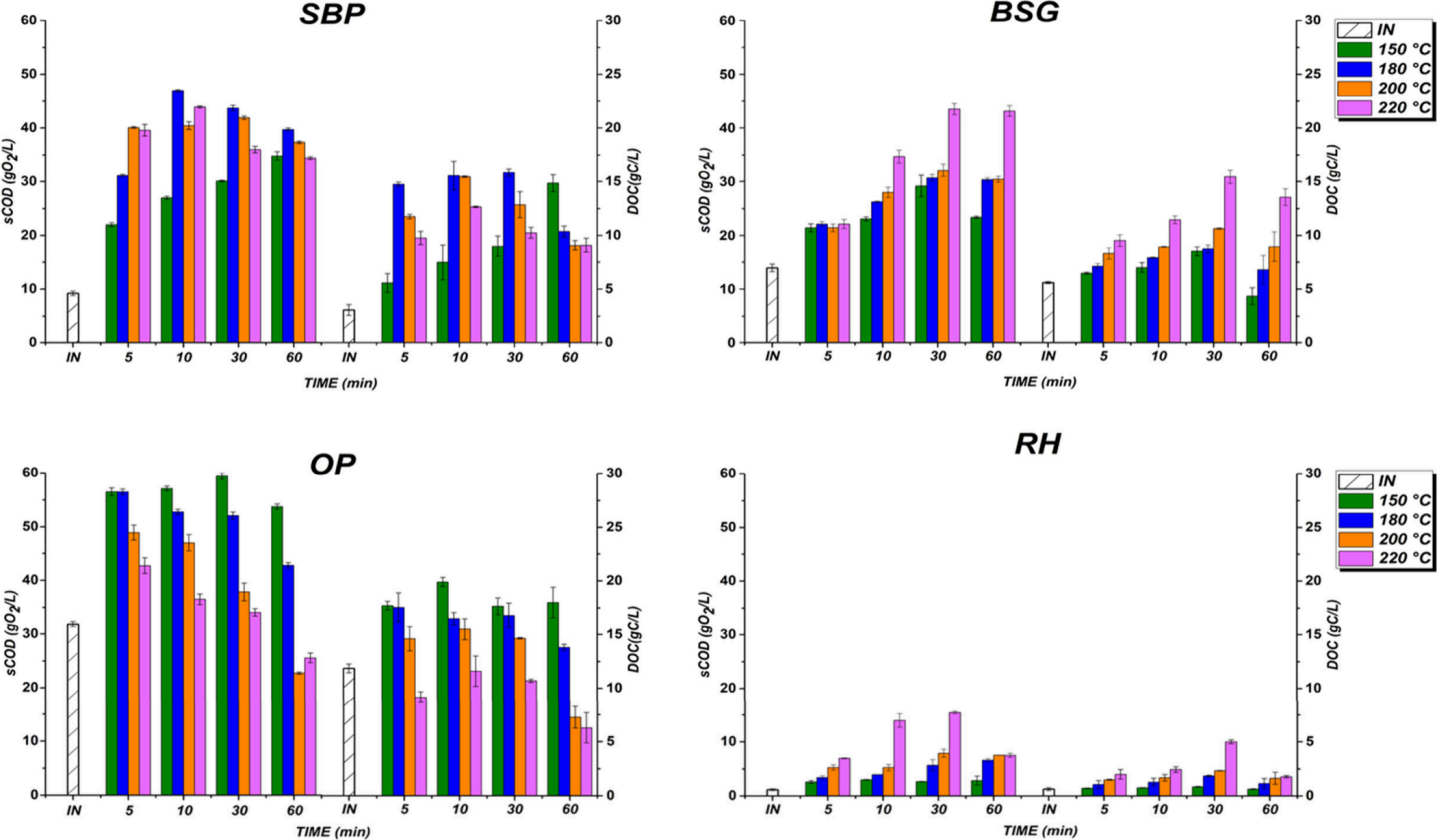
DOC and sCOD of SBP, BSG, OP, and RH by microwave-assisted pretreatment (MW). Note: IN—initail time.

MW pretreatment is particularly effective for achieving rapid hydrolysis, especially for substrates such as SBP and OP. Its efficiency lies in its ability to accelerate the breakdown process, making it ideal for scenarios requiring the quick release of soluble organic compounds.

SBP solubilization reached its highest values at 180 °C after 10 min, with sCOD and DOC concentrations of 47 g O_2_/L and 15.5 g C/L, respectively, but declined at 220 °C (39.7 g O_2_/L, 12.6 g C/L). Prolonged exposure further reduced the efficiency. The sCOD yield of 58.75% obtained in this study surpasses 13.7% of sCOD obtained by Ozkan et al. (2011), under MW pretreatment conditions (700 W, 170 °C) for 30 min. Moreover, it closely aligns with their 58% sCOD yield from thermal alkaline pretreatment at 121 °C.^29^ These comparisons underscore the impact of optimizing the microwave power and temperature conditions to enhance the solubilization efficiency.

BSG solubilization showed no significant changes during the first 5 min, with sCOD and DOC between 21.1–22.4 g O_2_/L and 6.5–9.5 g C/L. At 220 °C after 10 min, solubilization increased to 34.7 g O_2_/L sCOD and 11.4 g C/L DOC, reaching maximum values of 43.5 g O_2_/L and 15.4 g C/L after 30 min. Solubilization efficiency decreased with extended pretreatment durations.

OP showed fast initial solubilization across all temperatures, with the highest values at 180 °C (56.5 g O_2_/L sCOD, 17.5 g C/L DOC), followed by 200 °C (48.9 g O_2_/L, 14.6 g C/L) and 220 °C (42.7 g O_2_/L, 9.1 g C/L). Prolonged pretreatment periods reduced the solubilization yields, with the lowest values in terms of sCOD and DOC observed at 200 °C after 60 min (22.7 g O_2_/L, 7.2 g C/L). Consistent solubilization at 150 °C achieved maximum values of 59.4 g O_2_/L and 17.6 g C/L after 30 min, indicating this as an optimal condition for sustained OP solubilization.

RH solubilization remained consistently limited, achieving maximum sCOD and DOC concentrations of 15.4 g O_2_/L and 6 g C/L at 200 °C after 30 min. This is consistent with Kainthola et al. 2019, who reported 15000 mg/L sCOD for rice straw at 190 °C (1200 W).^30^ RH showing limited response highlights the need for alternative or supplementary methods to enhance solubilization efficiency.

The difference in fiber composition plays a crucial role in the efficiency of solubilization during the MW pretreatment. Specifically, SBP and OP exhibit a notably higher percentage of soluble fibers (42% in SBP and 66% in OP) along with significant quantities of carbohydrates and low lignin content (1.3% for OP and 3.5% for SBP). The low lignin proportion reduces structural resistance, facilitating an enhanced interaction between water molecules and the polysaccharide matrix under MW conditions. These attributes enable faster and more efficient hydrolysis at relatively lower temperatures.^31,32^

In fact, improved saccharification rates in OP after MW treatment have been already reported in the literature due to its carbohydrate-rich composition.^33–36^ The aforementioned studies highlight the effectiveness of MW pretreatment in optimizing the subsequent biomass conversion processes.

#### 3.2.2 Conventional Hydrothermal Reactor Solubilization

Figure 2 shows the solubilization efficiency for the four biomasses (SBP, OP, BSG, and RH) treated in a hydrothermal digester reactor (HTR) at 180, 200, and 220 °C for 30, 60, and 120 min.

**Figure 2:**
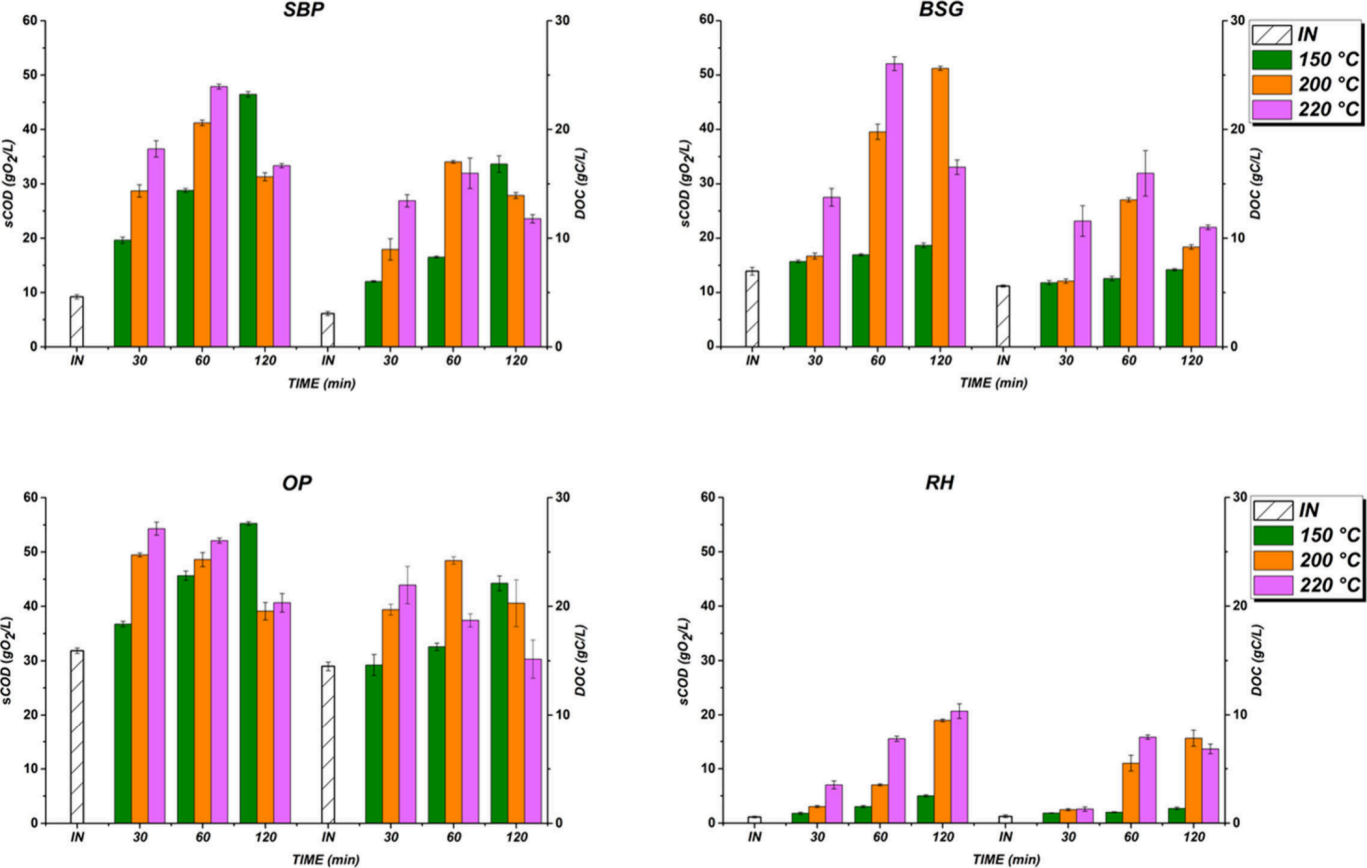
DOC and sCOD of SBP, BSG, OP, and RH by hydrothermal pretreatment (HTR). Note: IN—initial time.

At 180 °C, solubilization progressively improved, with SBP and OP exhibiting the highest efficiency. After 120 min, SBP and OP reached maximum sCOD and DOC values of 46.5 g O_2_/L, 16.8 g C/L, and 55.2 g O_2_/L, 22.1 g C/L, respectively. The solubilization yield of 61% achieved for OP at 150 °C under HTR aligns with previous findings;^37,38^ these authors reported yields of 46.6% of lemon peels and approximately 60% for orange peels, respectively, at 160 °C. Their study confirmed that this temperature improves the highest release of soluble organic compounds from biomass. In contrast, BSG and RH displayed lower yields, recording sCOD and DOC values of 18.6 g O_2_/L, 7.1 g C/L, and 5 g O_2_/L, 1.3 g C/L, respectively. At 200 and 220 °C, the solubilization yield improved across all substrates. SBP and OP reached their highest sCOD and DOC levels at 60 min (41.2 g O_2_/L, 17 g C/L) and 30 min (49.5 g O_2_/L, 19.7 g C/L), respectively, before declining. BSG and RH gradually increased, achieving their highest solubilization at 120 min, with BSG at 51.2 g O_2_/L, 9.2 g C/L, and RH at 19 g O_2_/L, 7.8 g C/L. Other studies have showed that temperatures above 200 °C are particularly effective in enhancing BSG degradation and bioproduct recovery.^39–41^

Overall, the temperature and time significantly influenced solubilization. Optimal hydrolysis occurred at 220 °C, with OP requiring only 30 min, and SBP and BSG 60 min, while RH showed the slowest and least effective solubilization.

The HTR method generally achieves higher solubilization efficiency over longer treatment times for substrates with complex structures such as BSG and RH, which are more resistant due to their significant lignin and lignocellulosic content. Specifically, BSG and RH contain significant lignin levels (7% and 14%, respectively). Additionally, there is a low soluble fiber content (38% for BSG and 16% for RH) compared to SBP and OP. Lignin acts as a barrier to hydrolysis, limiting the accessibility of hydrolytic agents to cellulose and hemicellulose fractions, while its interwoven structure and chemical bonds with hemicellulose further complicate degradation. Under HTR conditions, elevated temperatures disrupt hydrogen bonds within the lignocellulosic matrix and induce partial depolymerization of lignin, thereby exposing cellulose and hemicellulose for hydrolysis.

However, the efficiency of solubilization is highly dependent on the elevated temperatures and treatment duration. Studies indicate that high temperatures positively impact materials with substantial lignin content, as seen in RH. However, the prolonged treatment time may become a limiting factor, potentially decreasing solubilization efficiency due to the degradation of soluble compounds into recalcitrant byproducts such as furfural and hydroxymethylfurfural (HMF), which hinder further hydrolysis.

In Mussatto and coauthors’ works it was pointed out that the difficulty for the hydrolysis of BSG is due to its high lignin and hemicellulose content.^42,43^ In parallel, Lu et al. (2012) argued that the difficulty in the solubilization of RH is due to its significant lignocellulosic composition, which requires strict pretreatment conditions.^44^

Therefore, the choice of the pretreatment method and conditions should be tailored according to the specific biomass and the desired results for solubilization efficiency.

### 3.3 Impact of the Pretreatment Process on Sugar Production and Beyond

The effect of hydrothermal pretreatment on product distribution (TRS, PR, VFA and TP) was also assessed.

#### 3.3.1 Total Reducing Sugar (TRS)

The total reducing sugar (TRS), expressed in terms of grams TRS/L released to the aqueous phase after MW or HTR pretreatment, is shown in Figure 3.

**Figure 3:**
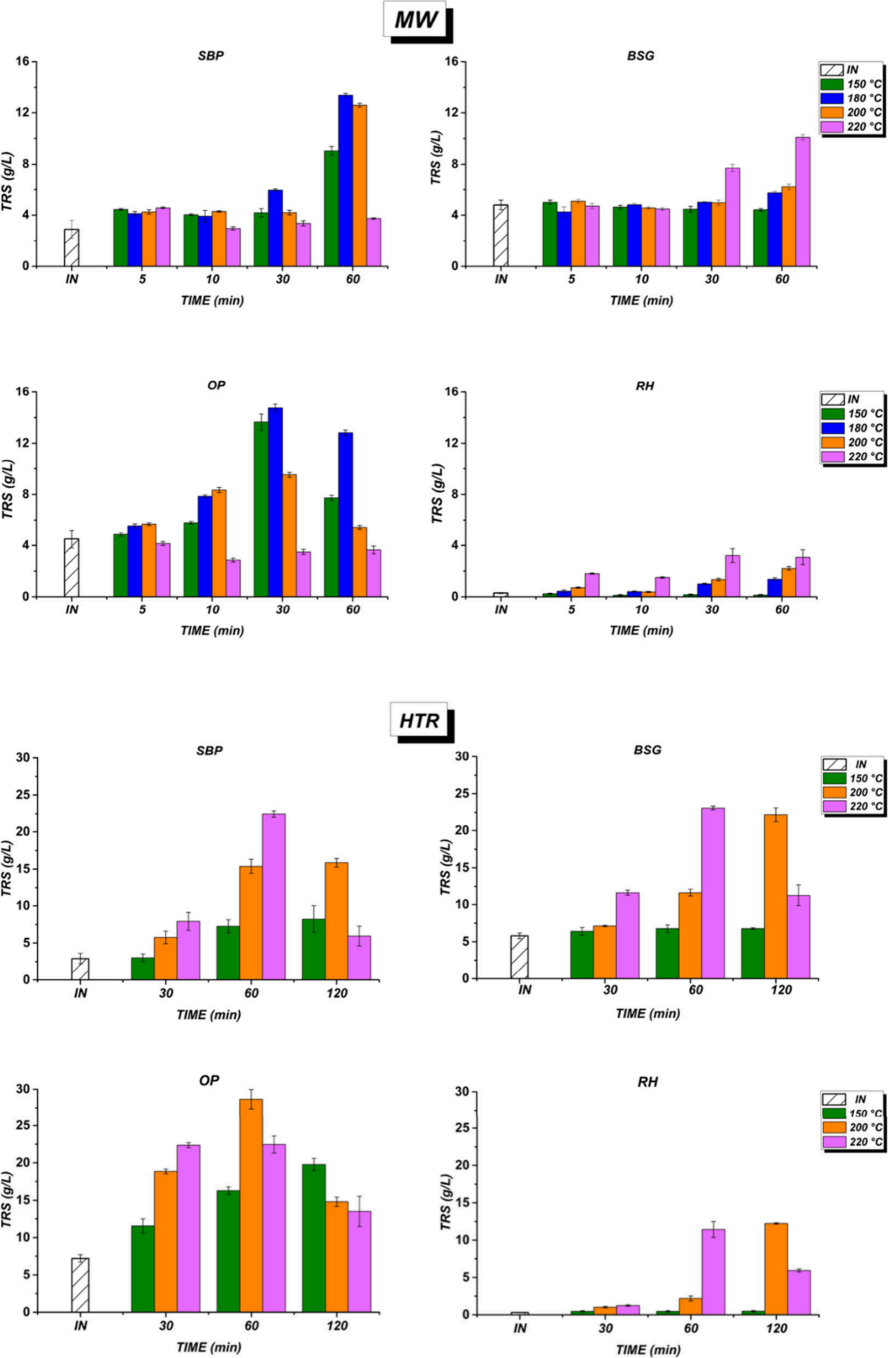
Total reducing sugar (TRS), expressed in terms of g/L, under a microwave-assisted (MW) and hydrothermal reactor (HTR). Note: IN—initial time.

TRS concentrations varied according to biomass composition. For MW pretreatment, SBP and OP reached their highest TRS concentrations at 180 °C, with 12 g/L after 60 min and 14 g/L after 30 min, respectively. BSG was achieved at 11 g/L at 220 °C after 60 min. RH had the lowest yield of 3.2 g/L at 220 °C after 60 min, indicating a lower hydrolysis efficiency.

TRS in OP declined significantly after 30 min, suggesting degradation or conversion of these monomers into other bioproducts, such as acids, or their transformation into new compounds. HTR showed a significant impact over TRS, increasing its value for all biomasses over time. The highest concentrations for SBP and BSG were 22 and 23 g/L at 220 °C after 60 min, though yields decreased afterward. At 200 °C, there was no significant effect on TRS for SBP, while BSG increased from 10 to 22 g/L after 120 min. This result aligns with Costa et al. (2014), who reported 12.15 g/L TRS from sugar cane bagasse under similar conditions.^45^ OP exhibited a notable response, reaching 18 g/L at 150 °C after 120 min and attaining a maximum of 28 g/L at 200 °C after 60 min before declining. At 220 °C, TRS stabilized around 22.4 g/L between 30–60 min, then decreased after 120 min. These results are consistent with Rivas et al. (2008), in which 38.2 g/L TRS from OP at 130 °C was founded.^46^ RH exhibited the lowest TRS concentration among the tested biomasses, reaching 12 g/L after 120 min at 200 °C. A similar yield was observed after 60 min at 220 °C. The hydrothermal pretreatment effectively decomposes lignocellulosic biomass, enhancing sugar release into the liquid phase. By breaking down hemicellulose and partially degrading cellulose at elevated temperatures, this method significantly improves TRS yields.^10^

#### 3.3.2 Total Protein Concentration (PR)

Figure 4 shows the effect of MW and HTR pretreatment on the protein’s extraction for the tested biomasses.

**Figure 4:**
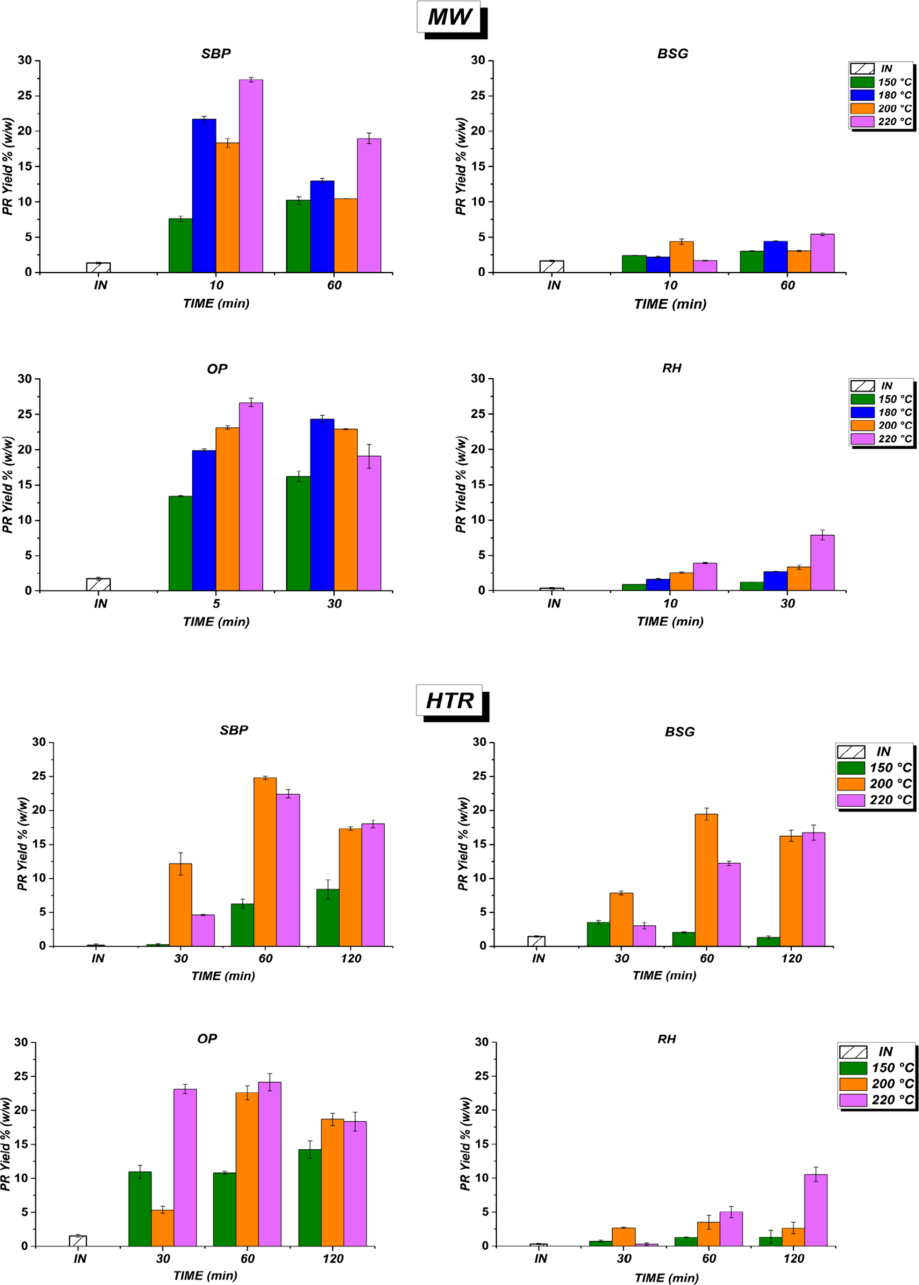
Total protein yield (PR), expressed in % (w/w), of SBP, BSG, OP, and RH under microwave assisted (MW) and hydrothermal reactor (HTR) pretreatments. Note: IN—initial time.

Total protein concentration was evaluated by using bovine serum albumin (BSA) as a standard to compare between the two processes.

For SBP and OP, similar protein yields were observed. Using the MW pretreatment, SBP yielded 21.8% PR after 10 min at 220 °C, while a slightly higher yield (22.42%) was obtained at the same temperature after 60 min. In the case of HTR pretreatment, the maximum PR yield (24.8%) was achieved with the OP at 200 °C for 60 min. However, the highest PR recovery of 26.3% was obtained under MW treatment at 220 °C after just 5 min.

These results may be explained by the biochemical composition of the substrates. SBP and OP contain more readily extractable soluble proteins and a less structurally rigid lignocellulosic matrix. This structure facilitates protein denaturation and unfolding during thermal or microwave treatments, increasing the extractability. Prolonged HTR exposure further enhances matrix disruption, partial lignin depolymerization, and polysaccharide hydrolysis, which promotes protein solubilization and the release of sugars that may contribute to VFA formation. However, MW pretreatment was less effective for another biomass, such as BSG and RH. In these cases, the maximum PR yields achieved were 6% and 7.9% after 60 and 30 min of MW treatment at 220 °C, respectively.

Under HTR conditions, the PR yield in BSG increased significantly, reaching 19.41% after 60 min at 200 °C, while in RH it only reached 10.55% after 120 min at 220 °C. The lower MW efficiency may be attributed to the complex fiber-encased structure and higher lignin content of BSG and RH, which limit protein accessibility. HTR, with its uniform and sustained thermal exposure, disrupts these matrices more effectively, allowing greater protein solubilization. Prolonged treatment, however, leads to a decrease in total protein, reflecting degradation into smaller molecules such as amino acids and peptides, contributing to the soluble organic fraction.

Literature supports these findings, for example Yin et al. (2014) from 30 g of food wastes (mainly rice, meat, vegetables and tofu) reported 22.50 g/kg of solubilized protein after 30 min of HTR pretreatment at 220 °C.^47^ Other authors as Qin et al. (2018) studied the hydrothermal pretreatment of BSG for protein extraction, achieving 66% of the total protein (14.91 g/100g raw BSG) successfully extracted at 60 °C after 24 h of treatment.^48^

According to the review of Scherzinger and Kaltschmitt (2021), the application of MW pretreatment to various food wastes at 175 °C has been successful for enhanced solubilization of proteins, sugars and humic-like substances.^49^ Overall, the results demonstrate that substrate composition strongly influences protein solubilization, which subsequently affects the performance of subsequent biomass hydrolysis and bioconversion processes.

#### 3.3.3 Effect of Hydrothermal Process in VFA Production

The total volatile fatty acid (TVFA) production in terms of acetic acid is shown in Figure 5. Within the tested conditions and selected substrates, the MW pretreatment does not seem to be effective for the TVFA production. Overall, the conversion of biomass wastes to TVFA is lower than 3 gH–Ac/L. Especially with RH and BSG, TVFA production is very low (0.5 g H–Ac/L), suggesting that the MW-assisted process has intrinsic limitations.

**Figure 5:**
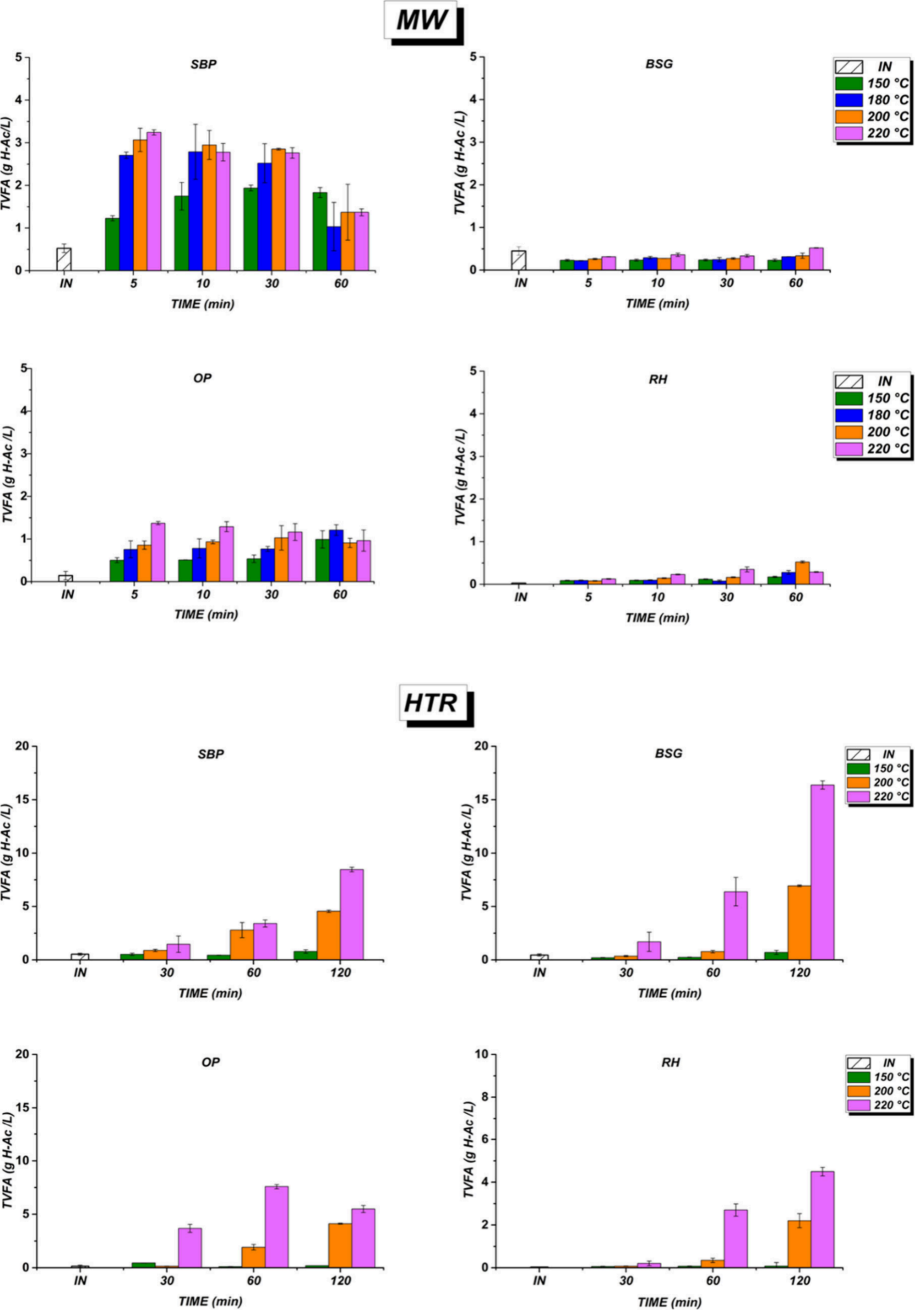
Total volatile fatty acids (TVFA), expressed as g H–Ac/L, for SBP, BSG, OP, and RH under microwave assisted (MW) and hydrothermal reactor (HTR) pretreatment. Note: IN—initial time.

The high silica and lignin content in RH and BSG probably rendered these materials resistant to MW pretreatment, thereby constraining the conversion of hydrolysates to VFA.^50^ SBP and subsequently OP showed the highest TVFA production after 5 min at 220 °C, with maximum values of 3.2 and 1.4 g of H–Ac/L, respectively. TVFA production remained steady for 30 min before decreasing at all temperatures. This suggests that while initial TVFA production is fast, conversion efficiency decreases over time due to possible depletion of readily available substrates or inhibition by accumulated byproducts. The lower lignin content and higher carbohydrate accessibility of SBP are more suitable for the MW pretreatment. Furthermore, pectin and essential oils of OP may hinder full hydrolysis and VFA conversion; despite relatively high initial sugar release, these components may slow overall process efficiency.^51,52^

Figure 5 also highlights the substantial impact of HTR pretreatment on VFA production, showing consistent increases over time at 200 and 220 °C. Maximum concentrations were achieved after 120 min at 220 °C: 16 g H–Ac/L for SBP, 8.5 g H–Ac/L for BSG, and 4.5 g H–Ac/L for RH. These results are similar to those obtained 20 g/L VFA from maize stalks under similar hydrothermal conditions (hydrothermal treatment severity HTS factor 5.16, 220 °C, oxygen-free).^53^ Similarly, Ziemiński et al. (2014) observed comparable trends for SBP, 1.6 mg/mL of VFA at 200 °C after 20 min after liquid hot water pretreatment.^54^ For OP, the highest TVFA concentration of 8 g/L was reached after only 60 min at 220 °C. However, after this maximum, the TVFA concentration gradually decreased, indicating that the optimal hydrothermal pretreatment time for OP is shorter than that for the other tested biomasses. Pretreatment at 150 °C was ineffective across all biomasses with VFA yields lower than 0.5 g/L, indicating insufficient conversion efficiency at lower temperatures. Acetic acid predominated under the MW pretreatment, comprising 97% of TVFA.

HTR pretreatment, however, released multiple acids: acetic acid accounted for 85% in RH, 45% in SBP, 42% in OP, and 12% in BSG; propionic acid represented 6% in RH, 36% in SBP, 12% in OP, and 80% in BSG; butyric acid made up 35% in OP and 18% in SBP, respectively. Herein, the above results demonstrate the diverse effectiveness of HTR pretreatment in the production of several VFAs at higher concentrations depending on the biomass used.

#### 3.3.4 Total Polyphenols (TP)

The extraction and quantification of polyphenols during pretreatment processes have garnered increasing attention, driven by their dual significance as high-value bioactive compounds for cosmetic applications and their role as fermentation inhibitors in downstream bioprocessing.^55,56^ This study measured total polyphenol (TP) concentrations using gallic acid as a standard (Figure 6). MW pretreatment yielded 0.2–4.2 g/L of polyphenols, with BSG and RH producing below 0.8 g/L. Maximum concentrations were observed in SBP and OP under optimal conditions: 3.8 g/L at 200 °C after 10 min for SBP, and 4.22 g/L at 220 °C after 30 min for OP. In contrast, Petrotos et al. (2021) achieved 8040 mg/L polyphenols from orange pomace using MW-assisted extraction (2,000 W, 80 °C, 10% liquid/solid ratio, 30 min).^57^

**Figure 6:**
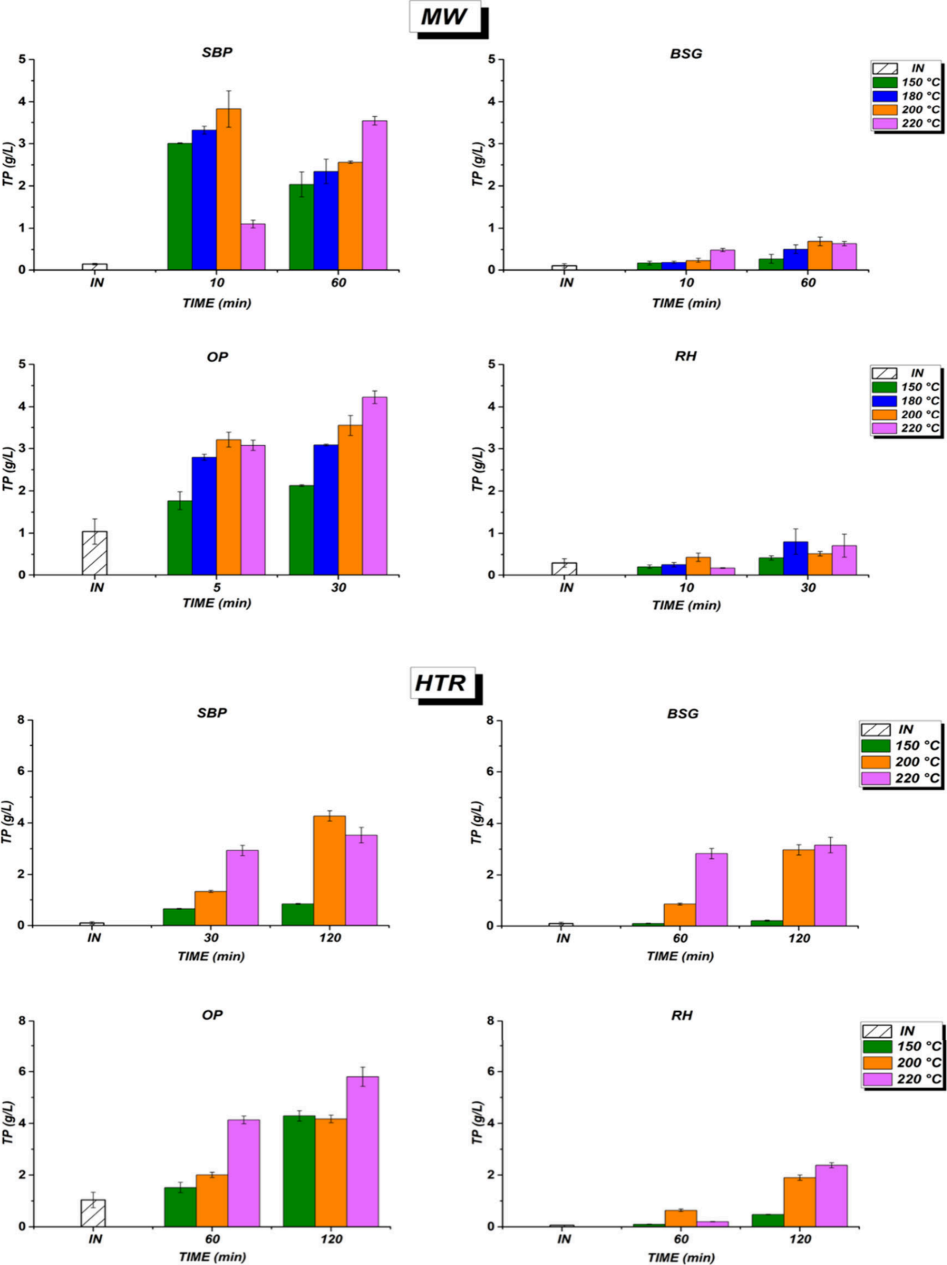
Total polyphenol concentration (TP), expressed as g/L, for SBP, BSG, OP, and RH under microwave assisted (MW) and hydrothermal reactor (HTR) pretreatments. Note: IN—initial time.

Hydrothermal reactor (HTR) pretreatment (Figure 6, second part) showed a maximum TP yield after 120 min: 5.8, 4.26, 3.56, and 2.38 g/L for OP, SBP, BSG, and RH, respectively. SBP reached its peak at 200 °C, while OP, BSG, and RH achieved maximums at 220 °C. This variation may be due to the substrate composition. SBP and OP contain higher levels of (NDF)-soluble fibers, which favor the phenolic compounds formation at high temperature. The lower content of (NDF) soluble fiber in RH and BSG results in a lower yield of polyphenols during pretreatment.

### 3.4 Comparative Study of the Pretreatment Methods

Figure 7 shows the relative yields of the product distributions corresponding to the two best performing biomass wastes.

**Figure 7:**
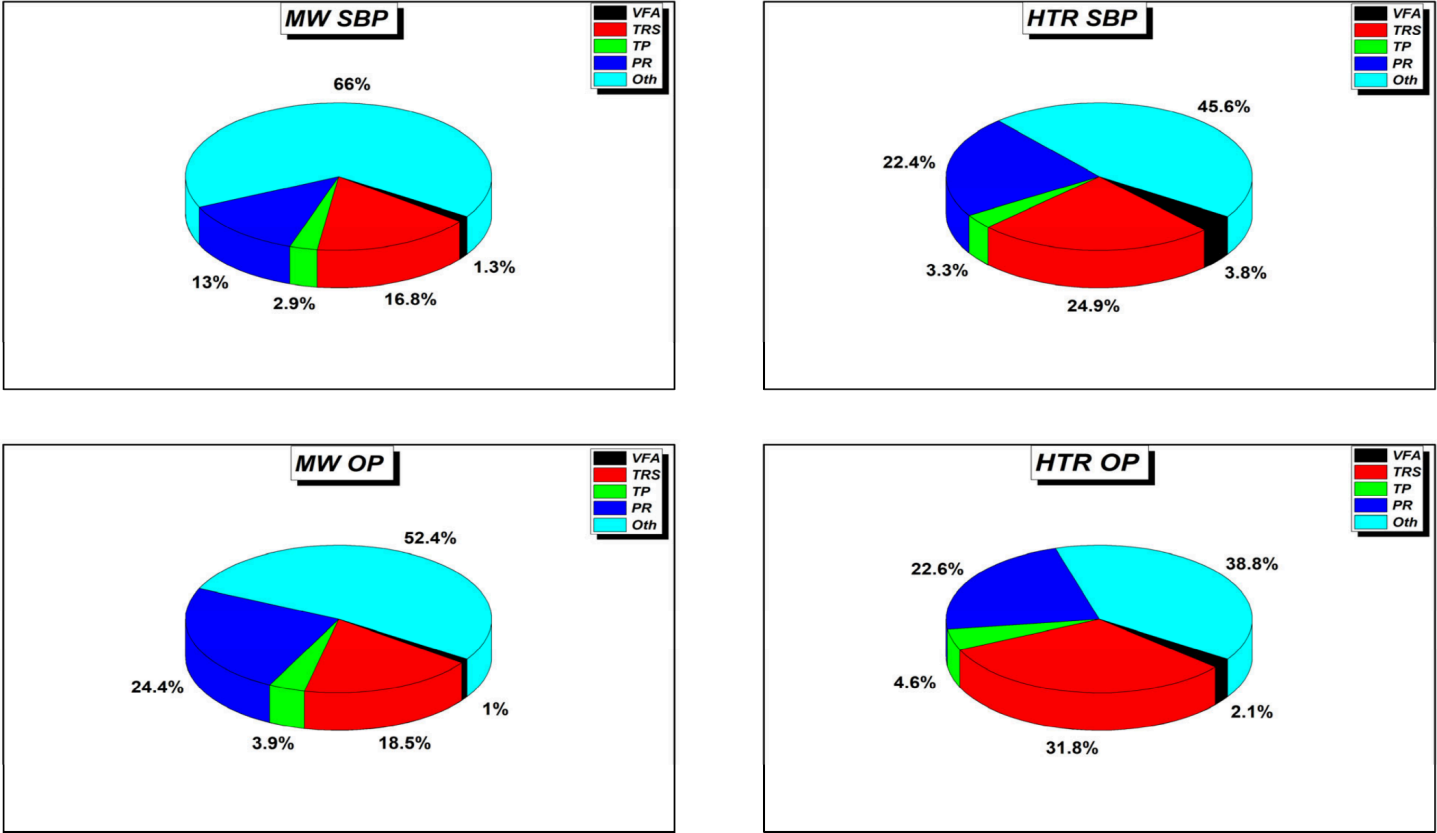
Percentage of the high added value bioproducts extracted from the solubilization of OP and SBP under microwave assisted (MW) and hydrothermal reactor (HTR) pretreatments.

For both pretreatments used, the fraction of unidentified undissolved biomass in the aqueous phase is denoted by “Oth”. The different parts of the solid biomass waste, such as cellulose and hemicellulose, dissolved as polymers and therefore tended to be reduced as sugar monomers, which could be assessed by TRS analysis. This suggests that all nondissolved biomass should not be presented in liquid phase. The selectivity percentages for these products during HTR exceeded those achieved with the MW pretreatment. This contrasts with the selectivity for other target products, specifically for TRS.

When comparing the two samples, both processes exhibit lower Oth values for the OP, which suggests that the higher proportion of OP polymer facilitates their conversion into simple, soluble polymers. However, the solubility gap between the two methods is very small with respect to the OP versus the TRS. This could be due to the inclusion of more cellulose and hemicellulose fractions in the case of SBP. As for the polyphenols and TVFA there is a fast similar distribution with no more than 7% selectivity sum. Thus, the method efficiency for this study could be compared to the TRS produced and the double biomass. Thus, HTR demonstrates a slight advantage in terms of the overall solubilization and bioproduct yield. However, considering the temperature and operation time required to reach the maximum (200 and 220 °C at 60 min) for practical and real conditions in general, the efficiency of the process should be assessed by the process cost, which is directly related to the energy consumed to produce this amount of product or biomass hydrolysis.

### 3.5 Energy Consumption

The trends in energy consumption for the hydrothermal reactor (HTR) and MW-assisted pretreatment methods are shown in the Supporting Information (Table S2). Overall, the HTR consumes approximately 2-fold higher energy than the MW-assisted method, particularly at longer treatment durations and higher temperatures.

## 4 Conclusions

This study demonstrates the effectiveness of both MW-assisted and HTR pretreatments in organic matter solubilization and the extraction of valuable bioproducts using four different biomasses as raw materials. The results indicate that both methods can achieve significant hydrolysis levels and bioproduct release, with variations depending on biomass type, temperature, and pretreatment duration. For OP and SBP, MW-assisted pretreatment at 180 °C for shorter durations (30 to 60 min) was the most efficient in achieving high solubilization and bioproduct yields, with reducing sugar concentrations of 18.75 (g/g) and 16.75 (g/g), respectively. With these operational conditions, the energy consumption was relatively low (25.10 and 33.10 kJ/g for 30 and 60 min, respectively). For more recalcitrant biomasses with high lignin content, such as BSG and RH, the HRT was more effective, with optimal results obtained at higher temperatures (220 °C) and longer treatment times (60 to 120 min), yielding 25.64 (g/g) and 13.58 (g/g) of reducing sugars, respectively. In this case, higher energy consumptions were required, with values of 70.85 and 78.72 kJ/g for 60 and 120 min, respectively.

The study also highlights the role of temperature and operation time in the degradation of these biomasses, noting that longer treatment times can lead to the breakdown of proteins and sugars into smaller byproducts such as amino acids, VFA and polyphenols. These results suggest that pretreatment conditions can be optimized to improve the recovery of important bioproducts for subsequent biorefinery operations and convert biomass more efficiently.

Overall, HTR outperformed MW pretreatment in terms of total biomass solubilization and bioproduct yield, particularly for more recalcitrant substrates. However, the MW pretreatment remains a viable option for certain biomass types, offering fast processing with relatively high extraction efficiencies at lower temperatures. Further research is needed to refine these processes and tailor them to specific biomass feedstocks for industrial-scale applications in biorefineries.

## Supplementary Material



## Notes

Methods and results of the statistical analytical and energy consumption (PDF)
