# Supplementary material for: Unlocking the Potential of Lignocellulosic Biomass: Microwave and Hydrothermal Pretreatment to Improve the Production of High Value-Added Biorefinery Compounds
Source: Energy Fuels. 2025 Oct 10;39(42):20383–96. doi: 10.1021/acs.energyfuels.5c03953 (PMC12557203; doi:10.1021/acs.energyfuels.5c03953)
Supplement: Supplementary file 1 [file ef5c03953_si_001.pdf]

Supporting Information

Unlocking the potential of lignocellulosic biomass: Microwave and Hydrothermal pretreatment to improve the production of high value-added biorefinery compounds

Amer Rouabhia [1,4], Carlos José Álvarez-Gallego [1,3], Luis Alberto Fernández-Güelfo [2,3 (*)], Mariana Valdez Castillo [4], Antonio Avalos Ramirez [4,5]

[1]University of Cádiz. Department of Chemical Engineering and Food Technology, Campus Puerto Real, 11510 Puerto Real, Cádiz, Spain
[2]University of Cádiz. Department of Environmental Technologies, Campus Puerto Real, 11510 Puerto Real, Cádiz, Spain
[3]Wine and Food Research Institute (IVAGRO), Campus Puerto Real, 11510 Puerto Real, Cádiz, Spain

[4]National Center in Environmental Technology and Electrochemistry, Shawinigan, G9N 6V8 Shawinigan (QC), Canada.
[5]Université de Sherbrooke. Department Chemical and Biotechnological Engineering, Faculty of Engineering, J1N 3C6 Sherbrooke (QC), Canada

E-mails by author order: amer.rouabhia@alum.uca.es; carlosjose.alvarez@uca.es; alberto.fdezguelfo@uca.es; mvcastillo@cnete.qc.ca; aaramirez@cnete.qc.ca.*

1. Statistical Analysis

This study used a linear mixed-effects model (Equation 3) to assess the impact of temperature and time on biomass solubilisation during two hydrothermal pretreatment processes. The analysis, performed in R software (v4.1) on a Linux platform, utilized the "*nlme*" library for model fitting and "*emmeans*" for post-hoc comparisons and marginal means. The model incorporated fixed effects (temperature, time) and random effects to account for biomass variability, enhancing the interpretation of interaction effects[1] .

$$log(Y) = \beta_0 + \beta_1\ substrate + \beta_2\ temperature + \beta_3\ time + u + \epsilon \qquad \textbf{Eq. (S1)}$$

- *log* (***Y***): Natural logarithm of parameters.
- ***β0***: Intercept.
- ***β1, β2, β3***: Coefficients for fixed effects (substrate, temperature, time).
- ***μ***: Random effect for different biomass samples.
- **ϵ**: Residual error term.

Table S1 presents the statistical p-values derived from the mixed model analysis using Equation S1, demonstrating significant effects of time and temperature ($p < 0.05$) on solubilisation parameters, including sCOD, DOC, TRS, VFA, TP, and PR. The findings indicate a distinct relationship between operational conditions (time and temperature) and solubilisation efficiency, underscoring the pivotal role of these factors in process optimization. This analysis provides valuable insights for identifying the optimal conditions required to achieve maximum solubilisation efficiency in the biomasses under study.

Overall, the majority of solubilization parameters exhibited significant p-values under both pretreatments. However, the impact of time on DOC (p value 0.330) and temperature on TRS (p value 0.820) under MW treatment was not significant. The statistical analysis aimed to assess the influence of various factors by considering interactions between different substrates. The insignificance of p-values for DOC and TRS is probably due to their extremely low concentrations in the tested biomasses, which may have constrained the measurable impact of time and temperature. These trends are further discussed and illustrated in the results section.

Table S1. *Statistical analysis of solubilisation parameters.*

	MW		***HTR***	
	Factor		***Factor***	
Variable	***Temperature P- value***	***Time P- value***	***Temperature P- value***	***Time P- value***
sCOD	*0.028*	*0.016*	*0.0163*	*0.0003*
DOC	*0.020*	***0.330***	*0.0072*	*0.0231*
TRS	***0.820***	*0.000*	*0.0035*	*0.0371*
VFA	*0.004*	*0.0014*	*0.0001*	*0.0001*
TP	*0.006*	*0.003*	*0.0007*	*0.0000*
PR	*0.000*	*0.022*	*0.0033*	*0.0010*

2. Energy consumption

Energy consumption data for the MW-assisted pretreatment are reported in the (Table S2). For this method, energy consumption increased progressively with temperature and time, starting at 8 kJ/g after 5 min at 150 °C and reaching a maximum of 40.14 kJ/g after 120 min at 220 °C. In contrast, the HTR consumed significantly more energy, with values of 51.17 kJ/g after 30 min, 70.85 kJ/g after 60 min and a maximum of 78.72 kJ/g after 120 min. This comparison highlights the higher energy requirement of the HTR, particularly at longer treatment times and higher temperatures, compared to the MW-assisted method, which consumes less energy even under longer conditions.

Table S2. *Energy consumption of MW pretreatment.*

E (kJ/g)	***Temperature (ºC)***			
Time (min)	***150***	***180***	***200***	***220***
5	*8.73*	*12.01*	*12.10*	*12.78*
10	*10.83*	*13.12*	*13.77*	*14.90*
30	*16.90*	*25.10*	*26.71*	*26.86*
60	*28.27*	*33.10*	*36.82*	*40.14*

These consumptions have been calculated applying equation (S2).

Energy equation: $E = \frac{P \times t}{m} \, (kJ/g)$ ***Eq. (S2)***

P: is the power of the device (in kW)

t: is the time of treatment (in seconds)

$\boldsymbol{m}$: is the mass of the biomass treated (in grams)

In the case of MW, P is calculated directly by the equipment. In the case of HTR, P is calculated according to equation (S3):

$$Pa = \frac{m \times Cp \times \Delta T}{t} \quad (W)$$ ***Eq. (S3)***

2

Where:

Pa: is the absorbed power (W)

m: is the total mass of the mixture (g)

Cp: is the calorific capacity of the mixture (J·g^{-1}·°C)

ΔT: is the variation of temperature (°C) during the chosen time t (s)

In the studied case, the mass in the reactor was a mixture of liquid and solid fractions. The Cp was calculated by proportionality from the Cpl of the liquid and the Cps of the substrate, Cpv of the vessel and Cpt of PTFE cup (equation S4)

$$Cp = \frac{liquid\ mass\ \times Cpl + Substarte\ mass\ \times Cps + vessel\ mass \times Cpv + PTFE\ cup\ mass \times Cpc}{Total\ mixture\ \ mass} \quad (J \cdot g^{-1} \cdot °C)$$

Eq. (S4)

Where Cp values are:

Cp liquid (water): 4.18 (J·g^{-1}·°C)

Cp substrate: 1.8 (J·g^{-1}·°C)

Cp vessel: 0.5 (J·g^{-1}·°C)

Cp Cup: 1 (J·g^{-1}·°C).

References

(1) Gałecki, A., Burzykowski, T. Linear Mixed-Effects Models Using R; New York,US, 2013; pp 245–273. https://doi.org/10.1007/978-1-4614-3900-4.

(2) Bichot, A.; Lerosty, M.; Radoiu, M.; Méchin, V.; Bernet, N.; Delgenès, J. P.; García-Bernet, D. Decoupling Thermal and Non-Thermal Effects of the Microwaves for Lignocellulosic Biomass Pretreatment. *Energy Convers. Manag.* **2020**, *203* (August 2019), 0–3. https://doi.org/10.1016/j.enconman.2019.112220.
